# 

**Published:** 1928

**Authors:** 


Vol. XLV. No. 170. WINTER, 1928.

### The Bristol Medico-Chirurgical Journal

PUBLISHED UNDER THE AUSPICES OF

THE BRISTOL MEDICO-CHIRURGICAL SOCIETY.

*Editor:* J. A. NIXON, C.M.G., M.D., F.R.C.P.

WITH WHOM ARE ASSOCIATED

E. W. HEY GROVES, M.S., F.R.C.S., B.Sc.,

E. WATSON-WILLIAMS, M.C., Ch.M., F.R.C.S.E.,

A. E. ILES, O.B.E., M.B., B.S. Lond., F.R.C.S.

CAREY F. COOMBS, M.D., F.R.C.P., *Assistant Editor.*

*Editorial Secretary:* A. L. FLEMMING, M.B., B.Ch.

BRISTOL: J. W. ARROWSMITH LTD., 11 QUAY STREET.
LONDON: J. W. ARROWSMITH (LONDON) LTD., 6 UPPER BEDFORD PLACE RUSSELL SQUARE.

Price Three Shillings net.

